# A Light‐Activatable Photocaged Variant of the Ultra‐High Affinity ALFA‐Tag Nanobody

**DOI:** 10.1002/cbic.202200079

**Published:** 2022-04-27

**Authors:** Benedikt Jedlitzke, Henning D. Mootz

**Affiliations:** ^1^ Institute of Biochemistry Department of Chemistry and Pharmacy University of Muenster Corrensstr. 36 48149 Münster Germany

**Keywords:** genetic code expansion, nanobodies, photocaging, protein design, spatiotemporal control

## Abstract

Nanobodies against short linear peptide‐epitopes are widely used to detect and bind proteins of interest (POI) in fusion constructs. Engineered nanobodies that can be controlled by light have found very recent attention for various extra‐ and intracellular applications. We here report the design of a photocaged variant of the ultra‐high affinity ALFA‐tag nanobody, also termed ALFA‐tag photobody. *ortho*‐Nitrobenzyl tyrosine was incorporated into the paratope region of the nanobody by genetic code expansion technology and resulted in a ≥9,200 to 100,000‐fold impairment of the binding affinity. Irradiation with light (365 nm) leads to decaging and reconstitutes the native nanobody. We show the light‐dependent binding of the ALFA‐tag photobody to HeLa cells presenting the ALFA‐tag. The generation of the first photobody directed against a short peptide epitope underlines the generality of our photobody design concept. We envision that this photobody will be useful for the spatiotemporal control of proteins in many applications using cultured cells.

## Graphical abstract

**Tag control**: Photo‐activatable antibodies are a recent addition to the chemical biology toolbox that allow the manipulation of biological processes by antigen‐binding with unprecedented spatiotemporal control. We report the successful extension of our design concept of suitably photocaged nanobodies, termed photobodies, to the first short tag (13 amino acids) binding nanobody, the ultra‐high affinity ALFA nanobody, to open the door for more fine‐tuned control.

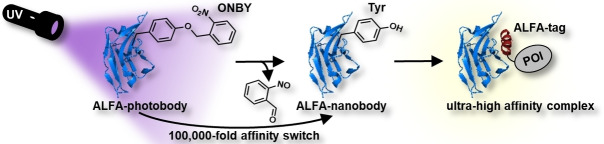

## Introduction

Antibodies that can be activated or switched by light or other stimuli have found rapidly increasing interest in recent years.^[1]^ On‐demand control of the exquisite binding affinity and selectivity of antibodies, antibody fragments or antibody‐like proteins towards their cognate epitopes offers many exciting possibilities in basic research and has potential applications in therapy.^[1]^ For example, activatable and switchable antibodies allow the spatiotemporal detection, masking and translocation of antigens in intracellular and extracellular settings, as well as the controlled delivery of drug conjugates and triggering of protein‐protein interactions. We and others have previously developed light‐activatable, photocaged nanobodies, also termed photobodies (Pb),^[2]^ for this purpose.[^2^, ^3^] Nanobodies (V_H_H; Nb) are single‐domain antibody fragments derived from heavy‐chain‐only antibodies found in *camelidae*.^[4]^ A single photo‐labile group on an amino acid centrally positioned in the paratope region was sufficient to decrease the binding affinity by up to 10,000‐fold^[2]^ for five different nanobodies directed against eGFP, EGFR and HER2.[^2^, ^3^] Antigen‐binding is restored by short irradiation with 365 nm, which removes the photo‐labile group and yields the native structure of the parent nanobody. Nanobodies are of particular interest for such conditional activation because among the many technical advantages they offer over IgGs, they can be easily fused with other proteins,^[4b]^ for example to create bispecific nanobodies for targeting and dimerizing two different antigens.^[2]^

We were interested to further test the generality of our photobody design concept and extend it to the first example of a photobody directed against a short, linear peptide epitope tag, namely the ALFA‐tag.^[5]^ Short peptide epitope‐tags like Myc‐, FLAG‐, HA‐ and His_6_‐tags and their specific full‐length IgG antibodies have been instrumental for a large variety of experimental techniques including immunostaining, fluorescence microscopy, immunoprecipitation, protein depletion and protein purification, for example.^[6]^ Compared to using antibodies raised against a native protein, such tags require the genetic fusion to the protein of interest. However, the targeting of fused epitope tags then features several advantages. It can be employed to detect POIs for which no antibody is available or which are of low immunogenicity.^[7]^ Tag‐directed antibodies are usually established to avoid cross‐reactions with other proteins in the proteome. Finally, compared to other fusion tags that have the size of a whole protein, like the fluorescent proteins, the short peptide‐epitope tags are considered beneficial as they have only minimal potential impact on protein behavior.^[8]^ As a result, short‐peptide epitope tags are widely used and are present in countless cloned constructs in research laboratories.

In recent years, an increasing number of nanobodies against short‐peptide epitopes has been reported, including the EPEA‐tag,^[9]^ BC2‐tag,^[10]^ myc‐tag,^[11]^ 6E‐tag,^[12]^ Moon‐tag,^[13]^ Pep‐tag^[14]^ and ALFA‐tag.^[5]^ Nanobodies offer several advantages over IgGs, such as a small size of only about 14 kDa, high stability, simple and comparably inexpensive recombinant production even in bacterial hosts, while rivaling IgGs with respect to antigen binding affinity and selectivity as well as displaying low immunogenicity to the human immune system.[^4b^, ^8a^]

The ALFA‐tag is a laboratory‐designed, amphiphilic peptide sequence (13 amino acids: SRLEEELRRRLTE) absent from common eukaryotic model systems and the human proteome. It is bound by the ALFA‐Nb with an ultra‐high affinity of *K_d_* =26 pM and has been successfully used in various extra‐ and intracellular applications.^[5]^ For these reasons, the ALFA system consisting of the ALFA‐tag and the ALFA‐Nb is being broadly used and adopted to numerous applications, e. g. in immunofluorescence^[15]^ or super‐resolution microscopy,^[5]^ immunoblotting[^5^, ^16^] and protein purification,[^5^, ^17^] and was the prime candidate for us to develop a photobody against a short epitope tag.

In previous work to design an activatable ALFA‐Nb, Farrants *et al*. achieved chemogenic control over binding the ALFA‐tag epitope inside mammalian cells by inserting a circularly permuted *Escherichia coli* dihydrofolate reductase (cpDHFR) into the ALFA‐Nb scaffold.^[18]^ They selected the region of the second complementarity determining region (CDR 2) as insertion site to modulate the binding affinity of the so‐called ligand‐modulated antibody fragment (LAMA). By adding trimethoprim (TMP) cpDHFR becomes stabilized and reduces its steric hindrance. However, this design strategy required testing of many insertion positions and revealed difficult‐to‐predict switching behavior in presence of ligands with activating, inhibiting or no effects at all. The best variant showed an about 15‐fold switch in binding affinities between the on and the off states.

An ALFA‐Nb controllable by light has not yet been reported. Other recent approaches to design light‐controllable nanobodies are based on fusion or insertion of photo‐responsive protein domains.^[19]^ However, also these approaches require extensive engineering of the protein structure with difficult‐to‐predict insertion sites and linker lengths. Furthermore, they do not reconstitute the native nanobody‐antigen complex when activated and thus suffer from increased molecular weights and generally from reduced binding affinities.

Here, we report the design, biochemical and cellular characterization of an ALFA‐Pb with an excellent on/off‐ratio in light activation. This is the first photobody directed against a short peptide‐epitope tag.

## Results and Discussion

**Figure 1:**
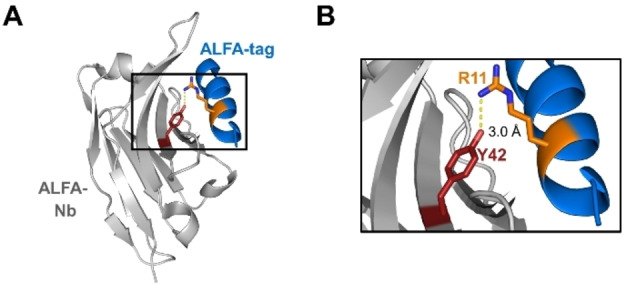
Considerations for photobody design. A) Schematic representation of the ALFA‐tag nanobody (Nb) binding the ALFA‐tag peptide (PDB: 6I2G).^[5]^ B) Close‐up view of tyrosine 42 (Y42) from the paratope region and its likely interaction with arginine 11 (R11) of the ALFA‐tag. For the ALFA‐Pb design the substitution Y42ONBY was introduced.

We aimed to develop a light‐activatable photobody variant of the ALFA‐Nb, i. e., a photocaged ALFA‐Nb. To apply the photobody design concept, we chose to replace a natural tyrosine residue in the paratope region of the ALFA‐Nb with the unnatural amino acid *ortho*‐nitrobenzyl tyrosine (ONBY), incorporated by the genetic code expansion technology.^[20]^ The steric hindrance of the photo‐labile *ortho*‐nitrobenzyl group (photocage group) was intended to perturb the protein‐protein interaction of the paratope‐epitope interface and hence to reduce binding affinity to the antigen. The ALFA‐Pb should then be activatable by irradiation with UV‐light (365 nm) to reconstitute the native nanobody sequence and therefore binding the antigen. Tyrosine is the most frequent amino acid in nanobody paratope regions.^[21]^ To our delight, the crystal structure of the ALFA‐Nb‐ALFA‐tag complex shows a tyrosine of the ALFA‐tag Nb that is in contact with the ALFA‐tag antigen (Figure 1A). Tyr42 is oriented towards Arg11 of the ALFA‐tag (Figure 1B). Despite this seemingly well‐defined molecular contact, Tyr42 sits rather on the edge of the paratope‐epitope interface, much less centrally located than tyrosine positions in other nanobodies that we had previously exploited for the design of photobodies.[^2^, ^3b^] Whether such a molecular arrangement with Tyr42 replaced by ONBY would still give rise to a sufficiently strong deactivation effect appeared as a challenging test for the generality of our photobody design strategy.

**Figure 2:**
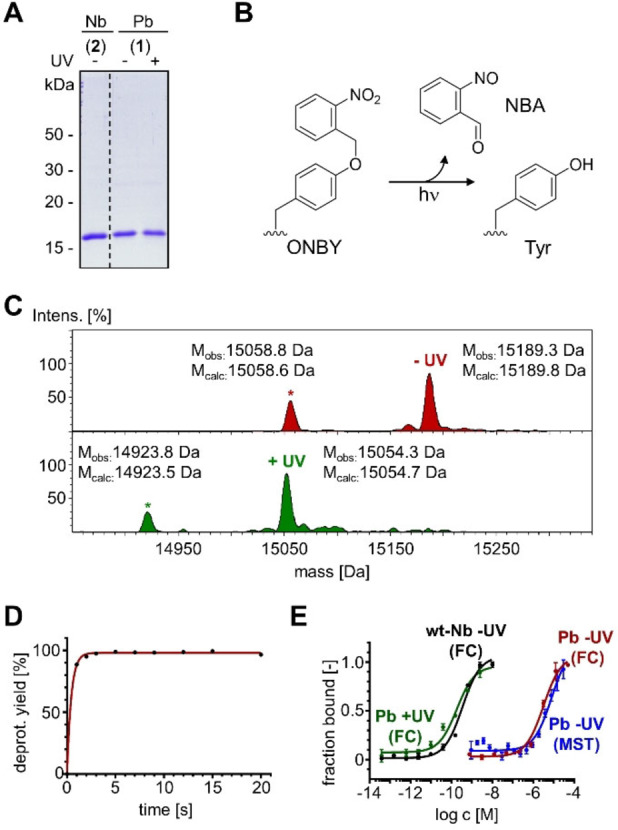
Characterization of the ALFA‐photobody (ALFA‐Pb) **1** and ALFA‐nanobody (ALFA‐Nb) **2**. A) Coomassie‐stained SDS‐PAGE gel showing purified proteins before (−) and after (+) exposure to UV light. B) Photodeprotection reaction of ONBY. NBA=nitrosobenzaldehyde. C) ESI‐MS analysis of **1** before (red) and after (green) photodeprotection with λ=365 nm. The asterisk labels protein species without start‐methionine. D) Time‐course of photodeprotection of **1** determined by ESI‐MS analysis. E) Determination of binding affinities of **1** by microscale thermophoresis (MST) and by flow cytometry (FC) of *E. coli* cells binding to sfGFP‐ALFA‐tag (**3**; see main text). See Figure S2 in the Supporting Information for FC histograms.

We produced the ALFA‐Pb (**1**) with the Tyr42ONBY mutation as a recombinant, C‐terminally His_6_‐tagged protein by periplasmic expression in *E. coli*, along with the wildtype ALFA‐Nb (**2**) for comparison. For ONBY incorporation an amber stop codon was introduced at position 42 by site‐directed mutagenesis of the encoding expression plasmid. The ONBY‐specific mutant of the *Methanococcus jannaschii* TyrRS and its cognate tRNA^CUA^ were encoded on a second plasmid.^[20b]^ Periplasmic expression reduces the potential partial reduction of the nitro group in ONBY to an amino group.^[22]^ To allow for simple site‐specific labeling, a single cysteine was appended close to the N terminus of both proteins. Figure 2A shows the proteins **1** and **2** analyzed on a Coomassie‐stained SDS‐PAGE gel, following purification by Ni‐NTA affinity and size exclusion chromatography.

To test photo‐decaging of the ONBY side chain according to Figure 2B, purified **1** was irradiated with UV light (365 nm, 530 mW, 5 cm distance) for 20 seconds. The conversion of the **1** to the restored wild‐type sequence was confirmed by mass spectrometry (MS) (Figure 2C). A time‐course analysis of photo‐deprotection revealed a virtually complete decaging after less than 5 seconds (Figure 2D).

To address the key question about the effect of the photocage group on binding affinity, we determined the dissociation constant *K_d_* for binding the ALFA‐tag before and after light activation. For microscale thermophoresis (MST) experiments, we prepared the fusion construct sfGFP‐ALFA‐tag (**3**) containing superfolder GFP (sfGFP) as a fluorescent protein marker. We incubated protein **3** at 10 nM concentration with a dilution series of photobody **1** and MST measurements revealed a *K_d_* of 7.9±0.1 μM. To corroborate this result by an independent method, we additionally determined the affinity by a previously developed protocol based on protein cell surface display and flow cytometry analysis.^[2]^ We mixed *E. coli* cells displaying the ALFA‐Pb, using the AIDA autodisplay system,^[23]^ with a dilution series of fluorescent sfGFP‐ALFA‐tag (**3**). The fluorescence of individual *E. coli* cells bound to **3** was determined by flow cytometry and this data was used to calculate a *K_d_* of 3.2±0.1 μM (Figure 2E; see Figure S2 in the Supporting Information for histograms).^[2]^ The consistent affinity in the single‐digit micromolar range determined by both methods thus showed a significant impact on binding of the photobody. Most importantly, these values suggest that a reduction in binding affinity of at least about 100,000‐fold was achieved, when compared to the *K_d_* of 26 pM reported for the wild‐type ALFA‐Nb.^[5]^ We then measured the binding affinity after photobody irradiation with UV light (365 nm). Due to an insufficient sensitivity of the MST instrument in the subnanomolar affinity regime this was only done with the flow cytometry assay. We irradiated *E. coli* cells displaying the ALFA‐Pb in 100 μL PBS buffer (OD600=1; 365 nm, 530 mW; 5 cm distance) and then added different concentrations of sfGFP‐ALFA‐tag (**3**), followed by flow cytometry analysis as described above. The calculated *K_d_* values were 183±6 pM for the decaged ALFA‐Pb and 346±5 pM for an unirradiated ALFA‐Nb control (Figure 2E). These numbers were about 7 and 13‐fold higher than the *K_d_* value reported for the ALFA‐Nb (26 pM).^[5]^ Given the difficulty to accurately measure such low *K_d_* values we believe to be on the edge of sensitivity with the experimental method, therefore our determined values might represent minimum values for a potentially even higher affinity. Potential influences from the surrounding sequence of the ALFA‐tag might be another explanation for the observed differences in affinity. In any case, photo‐decaging of the ALFA‐Pb clearly resulted in the desired effect with an impressive switch of binding affinity of at least about 9,200‐fold in the most conservative comparison of our determined values.

**Figure 3:**
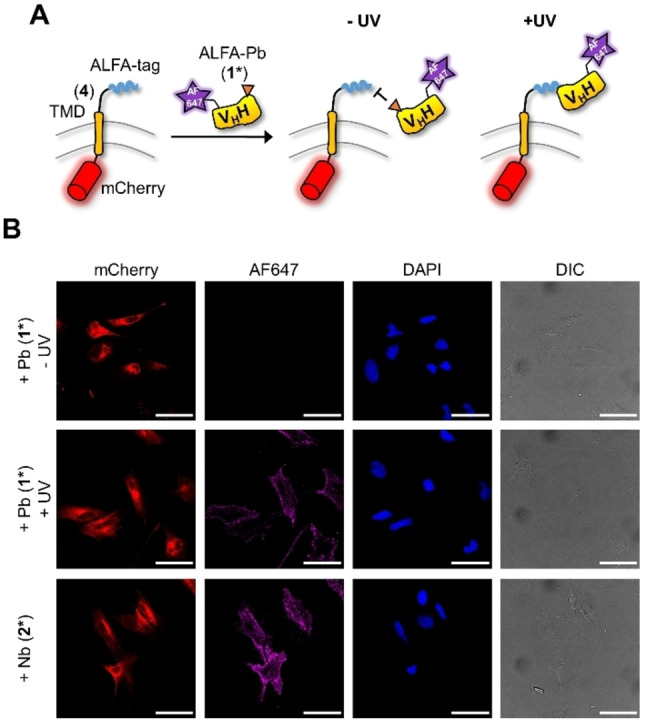
Extracellular binding assay of activated photobody. The ALFA‐tag photobody (Pb) 1* and control nanobody (Nb) 2* were bioconjugated with AlexaFluor647. A) Scheme of the assay using HeLa cells transiently transfected with ALFA‐tag‐TMD‐mCherry (**4**). B) Confocal fluorescent microscopy images of fixed cells and treated as illustrated in (A). Scale bar=50 μm; TMD=transmembrane domain.

We next investigated the engineered ALFA‐Pb in a binding assay on the surface of cultured mammalian cells (Figure 3A). We presented the ALFA‐tag on the cell surface of HeLa cells by transient transfection of a plasmid coding for a fusion protein with a signal peptide for protein export and further consisting of the ALFA‐tag followed by the transmembrane domain of the PDGF receptor (TMD) and a C‐terminal mCherry as a fluorescent protein (construct **4**). Of note, this TMD‐mCherry combination also gave rise to intracellular mCherry staining (Figure 3B), likely due to partial overloading of the secretory pathway or partial proteolysis as previously observed.^[24]^ We bioconjugated the ALFA‐Pb **1** and ALFA‐Nb control construct **2** with AlexaFluor647‐maleinimide at the unpaired cysteine to give proteins 1* and 2* (Figure S1). We then either treated 1* with UV light for photo‐decaging (λ=365 nm, 530 mW, 20 sec, 5 cm distance) or left it untreated in a control experiment. Each protein sample as well as control construct 2* was added to the growth medium of the transfected HeLa cells (final protein concentration 10 nM). Following incubation for 15 min at RT, cells were washed, fixed and analyzed by confocal laser scanning microscopy (CLSM). No binding of photocaged 1* to the cells was detected in the AF647 channel, neither for transfected nor for untransfected cells (Figure 3B, top row). In contrast, we observed specific binding to transfected cells when adding photo‐activated 1*, as intended (Figure 3B, middle row). The control experiment with uncaged ALFA‐Nb 2* showed specific binding of transfected cells independent of UV irradiation, as expected (Figure 3B, bottom row). Partial uptake of the AF647 label inside cells in both cases is likely explained by partial endocytosis of the receptor‐nanobody complex before fixing the cells. Together, these findings in combination with previous demonstrations of photocaged nanobodies[^2^, ^3^] suggest the potential of light‐activating the ALFA‐Pb in the context of cell culture experiments.

## Conclusion

We engineered an ALFA‐Pb by introducing a photocage group at the Tyr42 side chain of the parent ALFA‐Nb. Although Tyr42 is not centrally located in the paratope region, we show that the photocage decreases the binding affinity to the ALFA‐tag antigen by at least about 9,200 to 100,000‐fold compared to the parent, wild‐type nanobody.^[5]^ This surprisingly massive impact of a single substitution suggests a key contribution of Tyr42 to the binding affinity or a high cooperativity in the formation of the nanobody‐antigen binding interface. Similar to other photocaged proteins and nanobodies, UV irradiation (365 nm) restores the native protein within seconds. We show that this switch in binding affinity is in a useful range for cell culture experiments when addressing the ALFA‐tag as an extracellular epitope. Although not reported here, we and others have previously demonstrated that nanobodies photocaged with the same chemistry of the ONBY‐approach can be activated when binding to or when being located inside mammalian cells, by directly irradiating the cells for a few seconds, thus suggesting the same to be feasible for the ALFA‐Pb.[^2^, ^3b^, ^25^] The ALFA‐Pb represents the sixth example of a successful photobody design that so far only required a properly positioned tyrosine in the paratope region, thereby underlining the simplicity and generality of the approach. The nanobody backbone and fold remains unaltered and should therefore not be compromised by our approach. Further extensions of the photocage concept are conceivable to enlarge the versatility as well as the range of suitable nanobodies, antibodies and antibody‐like fragments. These include the utilization of other caged groups with sensitivity at longer wavelengths,^[2]^ caging other amino acid side chains, and considering mutations in the paratope region to accommodate non‐native residues that have only little impact on binding,^[26]^ but allow the addition of a photocage group. Importantly, the present work reports the first photobody directed against a short peptide‐epitope tag. Given the general usefulness of such epitope tags and the rapidly increasing utilization of the ALFA‐tag in particular, we envision the ALFA‐Pb to become a valuable tool to spatially and temporally control binding to its antigen in a wide range of experimental settings.

## Conflict of interest

The authors declare no conflict of interest.

1

## Supporting information

As a service to our authors and readers, this journal provides supporting information supplied by the authors. Such materials are peer reviewed and may be re‐organized for online delivery, but are not copy‐edited or typeset. Technical support issues arising from supporting information (other than missing files) should be addressed to the authors.

Supporting Information

## Data Availability

The data that support the findings of this study are available from the corresponding author upon reasonable request.

## References

[1] R. Lucchi , J. Bentanachs , B. Oller-Salvia , ACS Cent. Sci. 2021, 7, 724–738. 34079893 10.1021/acscentsci.0c01448 PMC8161478

[2] B. Jedlitzke , Z. Yilmaz , W. Dorner , H. D. Mootz , Angew. Chem. Int. Ed. 2020, 59, 1506–1510; 10.1002/anie.201912286 PMC7004160 31755215

[3a] T. Bridge , S. A. Shaikh , P. Thomas , J. Botta , P. J. McCormick , A. Sachdeva , Angew. Chem. Int. Ed. 2019, 58, 17986–17993; 10.1002/anie.201908655 PMC6973043 31609054

[3b] B. Jedlitzke , H. D. Mootz , ChemPhotoChem 2021, 5, 22–25.

[4a] C. Hamers-Casterman , T. Atarhouch , S. Muyldermans , G. Robinson , C. Hamers , E. B. Songa , N. Bendahman , R. Hamers , Nature 1993, 363, 446–448; 8502296 10.1038/363446a0

[4b] S. Muyldermans , Annu. Rev. Biochem. 2013, 82, 775–797. 23495938 10.1146/annurev-biochem-063011-092449

[5] H. Gotzke , M. Kilisch , M. Martinez-Carranza , S. Sograte-Idrissi , A. Rajavel , T. Schlichthaerle , N. Engels , R. Jungmann , P. Stenmark , F. Opazo , S. Frey , Nat. Commun. 2019, 10, 4403. 31562305 10.1038/s41467-019-12301-7 PMC6764986

[6] J. W. Jarvik , C. A. Telmer , Annu. Rev. Genet. 1998, 32, 601–618. 9928493 10.1146/annurev.genet.32.1.601

[7] B. Brizzard , BioTechniques 2008, 44, 693–695. 18474046 10.2144/000112841

[8a] R. W. Cheloha , T. J. Harmand , C. Wijne , T. U. Schwartz , H. L. Ploegh , J. Biol. Chem. 2020, 295, 15307–15327; 32868455 10.1074/jbc.REV120.012960 PMC7650250

[8b] C. Kuey , G. Larocque , N. I. Clarke , S. J. Royle , J. Cell Sci. 2019, 132. 10.1242/jcs.234955 PMC6857592 31601614

[9] E. J. De Genst , T. Guilliams , J. Wellens , E. M. O′Day , C. A. Waudby , S. Meehan , M. Dumoulin , S. T. Hsu , N. Cremades , K. H. Verschueren , E. Pardon , L. Wyns , J. Steyaert , J. Christodoulou , C. M. Dobson , J. Mol. Biol. 2010, 402, 326–343. 20620148 10.1016/j.jmb.2010.07.001

[10] M. B. Braun , B. Traenkle , P. A. Koch , F. Emele , F. Weiss , O. Poetz , T. Stehle , U. Rothbauer , Sci. Rep. 2016, 6, 19211. 26791954 10.1038/srep19211 PMC4726124

[11] J.-J. Li , F. Xu , Y.-W. Ji , M. Shu , Z. Tu , J.-H. Fu , China Biotechnol. 2018, 38, 61–67.

[12] J. J. Ling , R. W. Cheloha , N. McCaul , Z. Y. J. Sun , G. Wagner , H. L. Ploegh , Mol. Immunol. 2019, 114, 513–523. 31518855 10.1016/j.molimm.2019.08.008 PMC6774866

[13] S. Boersma , D. Khuperkar , B. M. P. Verhagen , S. Sonneveld , J. B. Grimm , L. D. Lavis , M. E. Tanenbaum , Cell 2019, 178, 458–472 e419. 31178119 10.1016/j.cell.2019.05.001 PMC6630898

[14] B. Traenkle , S. Segan , F. O. Fagbadebo , P. D. Kaiser , U. Rothbauer , Sci. Rep. 2020, 10, 14267. 32868807 10.1038/s41598-020-71091-x PMC7459311

[15a] N. Matsumoto , Y. Nemoto-Sasaki , S. Oka , S. Arai , I. Wada , A. Yamashita , J. Biol. Chem. 2021, 297, 100851; 34089703 10.1016/j.jbc.2021.100851 PMC8234217

[15b] M. A. Vigano , C. M. Ell , M. M. M. Kustermann , G. Aguilar , S. Matsuda , N. Zhao , T. J. Stasevich , M. Affolter , G. Pyrowolakis , Development 2021, 148, dev191700. 33593816 10.1242/dev.191700 PMC7990863

[16] B. M. Esch , S. Limar , A. Bogdanowski , C. Gournas , T. More , C. Sundag , S. Walter , J. J. Heinisch , C. S. Ejsing , B. Andre , F. Frohlich , PLoS Genet. 2020, 16, 1008745. 10.1371/journal.pgen.1008745 PMC7478846 32845888

[17] E. Perego , S. Reshetniak , C. Lorenz , C. Hoffmann , D. Milovanovic , S. O. Rizzoli , S. Koster , Sci. Rep. 2020, 10, 21086. 33273508 10.1038/s41598-020-77887-1 PMC7713060

[18] H. Farrants , M. Tarnawski , T. G. Muller , S. Otsuka , J. Hiblot , B. Koch , M. Kueblbeck , H. G. Krausslich , J. Ellenberg , K. Johnsson , Nat. Methods 2020, 17, 279–282. 32066961 10.1038/s41592-020-0746-7

[19a] D. Yu , H. Lee , J. Hong , H. Jung , Y. Jo , B. H. Oh , B. O. Park , W. D. Heo , Nat. Methods 2019, 16, 1095–1100; 31611691 10.1038/s41592-019-0592-7

[19b] A. A. Gil , C. Carrasco-Lopez , L. Y. Zhu , E. M. Zhao , P. T. Ravindran , M. Z. Wilson , A. G. Goglia , J. L. Avalos , J. E. Toettcher , Nat. Commun. 2020, 11, 4044; 32792536 10.1038/s41467-020-17836-8 PMC7426870

[19c] C. Carrasco-Lopez , E. M. Zhao , A. A. Gil , N. Alam , J. E. Toettcher , J. L. Avalos , Nat. Commun. 2020, 11, 4045; 32792484 10.1038/s41467-020-17837-7 PMC7427095

[19d] L. He , P. Tan , Y. Huang , Y. Zhou , Adv. Biol. 2021, 5, e2000541. 10.1002/adbi.202000541 PMC8295464 34028213

[20a] C. C. Liu , P. G. Schultz , Annu. Rev. Biochem. 2010, 79, 413–444; 20307192 10.1146/annurev.biochem.052308.105824

[20b] A. Deiters , D. Groff , Y. Ryu , J. Xie , P. G. Schultz , Angew. Chem. Int. Ed. 2006, 45, 2728–2731; 10.1002/anie.200600264 16548032

[21] C. McMahon , A. S. Baier , R. Pascolutti , M. Wegrecki , S. Zheng , J. X. Ong , S. C. Erlandson , D. Hilger , S. G. F. Rasmussen , A. M. Ring , A. Manglik , A. C. Kruse , Nat. Struct. Mol. Biol. 2018, 25, 289–296. 29434346 10.1038/s41594-018-0028-6 PMC5839991

[22] J. K. Böcker , W. Dorner , H. D. Mootz , Chem. Commun. 2019, 55, 1287–1290. 10.1039/c8cc09204d 30633261

[23a] J. Jose , R. M. Maas , M. G. Teese , J. Biotechnol. 2012, 161, 92–103; 22569038 10.1016/j.jbiotec.2012.04.001

[23b] S. Oloketuyi , C. Dilkaute , E. Mazzega , J. Jose , A. de Marco , Appl. Microbiol. Biotechnol. 2019, 103, 4443–4453. 30989251 10.1007/s00253-019-09823-x

[24] T. Dhar , H. D. Mootz , Chem. Commun. 2011, 47, 3063–3065. 10.1039/c0cc04172f 21234473

[25] E. F. Joest , C. Winter , J. S. Wesalo , A. Deiters , R. Tampe , Chem. Sci. 2021, 12, 5787–5795. 35342543 10.1039/d1sc01331a PMC8872839

[26a] S. B. Gunnoo , H. M. Finney , T. S. Baker , A. D. Lawson , D. C. Anthony , B. G. Davis , Nat. Commun. 2014, 5, 4388; 25073737 10.1038/ncomms5388 PMC4124856

[26b] H. Zhang , Y. Han , Y. Yang , F. Lin , K. Li , L. Kong , H. Liu , Y. Dang , J. Lin , P. R. Chen , J. Am. Chem. Soc. 2021, 143, 16377–16382. 34596400 10.1021/jacs.1c08521

