# Supplementary material for: A Light‐Activatable Photocaged Variant of the Ultra‐High Affinity ALFA‐Tag Nanobody
Source: Chembiochem. 2022 Apr 27;23(12):e202200079. doi: 10.1002/cbic.202200079 (PMC9324849; doi:10.1002/cbic.202200079)
Supplement: Supplementary file 1 — Supporting Information [file CBIC-23-0-s001.pdf]

ChemBioChem

Supporting Information

A Light-Activatable Photocaged Variant of the Ultra-High Affinity ALFA-Tag Nanobody

Benedikt Jedlitzke and Henning D. Mootz*

Table of contents

Supporting Figures	page S2
Materials and Methods with Supporting Tables	page S4
Supporting Tables and Sequences	page S8
Supporting References	page S9
Appendix	page S9

SUPPORTING FIGURES

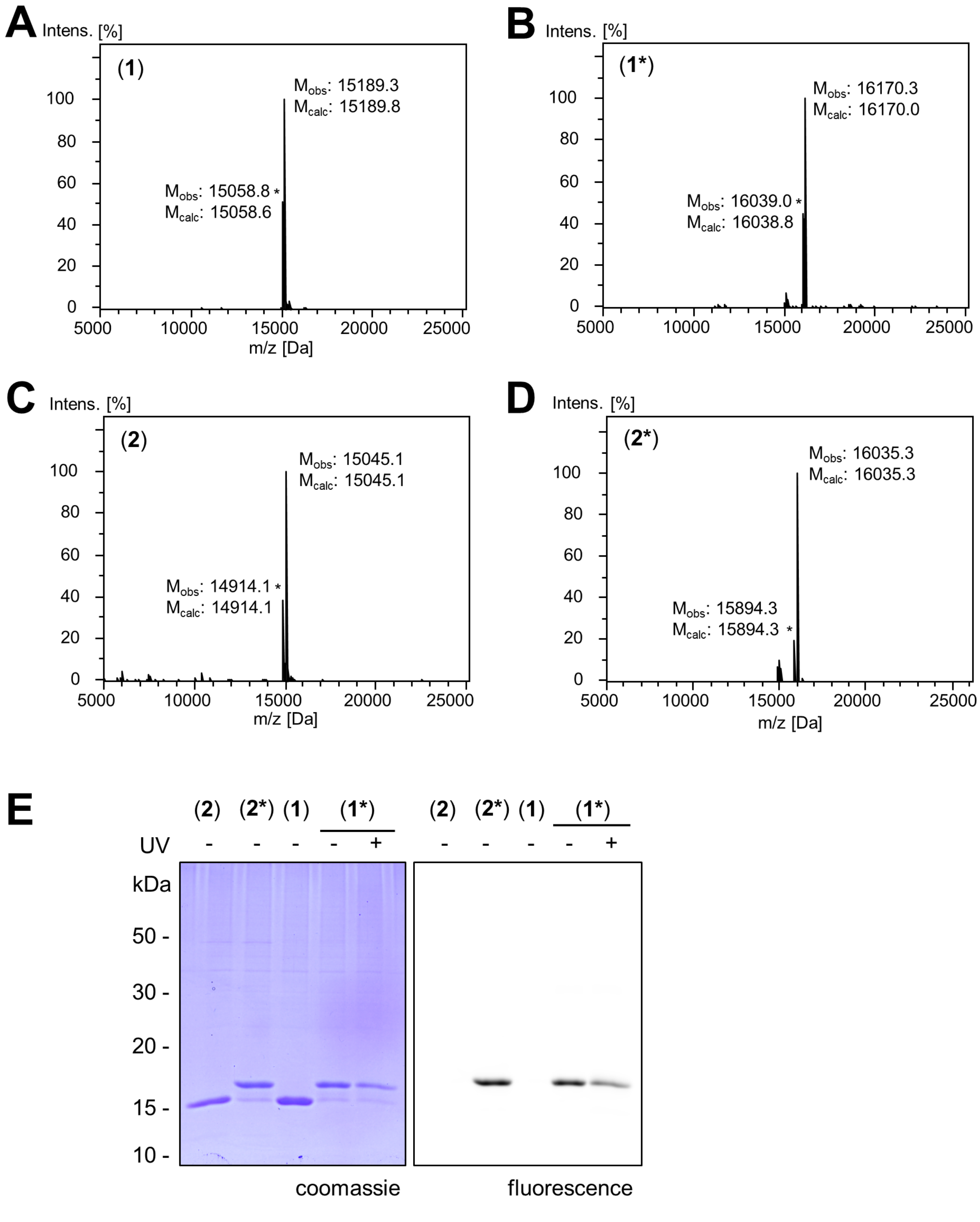

Figure S1. Further characterization of purified ALFA-photobody (**1**) and ALFA-nanobody (**2**) proteins. Proteins were analyzed either in AlexaFluor647-conjugated (*) or unconjugated form. (A - D) ESI-MS analysis of the indicated proteins. The asterisk labels protein species without start-methionine. (E) Analysis of the proteins on a Coomassie-stained SDS-PAGE gel and under UV illumination.

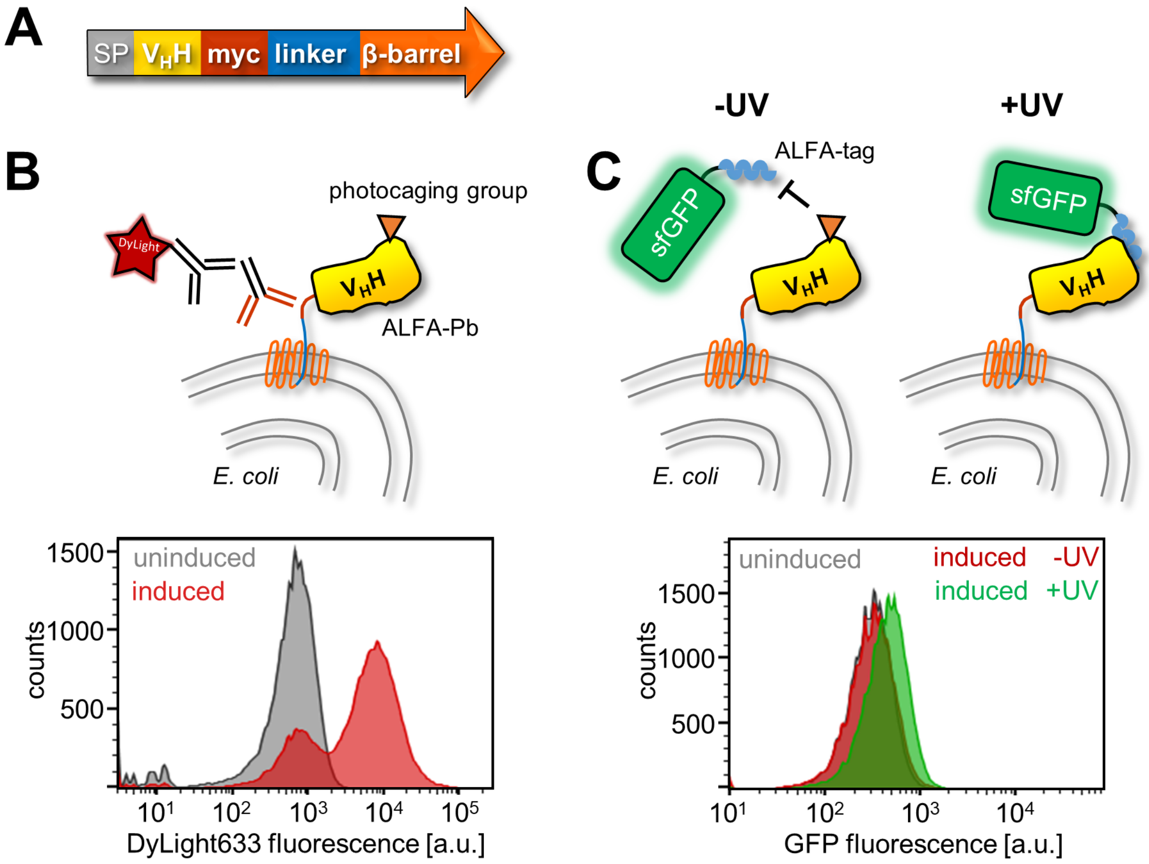

Figure S2. Display of ALFA-Pb on *E. coli* cell surface. A) Genetic fusion of ALFA-Pb with outer membrane protein of the AIDA autodisplay system.[1] SP = signal peptide. B) Upper panel: Scheme of ALFA-Pb displayed on *E. coli* cell surface and binding of primary and secondary antibody with DyLight633 fluorophore to the myc-tag to detect cells displaying protein. Lower panel: Flow cytometry analysis of *E. coli* cells presenting ALFA-Pb after incubation with DyLight633 coupled anti-myc antibody. Uninduced/induced refers to the induction of protein expression with IPTG. C) Upper panel: Scheme of ALFA-Pb displayed on *E. coli* cell surface and binding of sfGFP-ALFA-tag (**3**) after irradiation with light (+UV, λ=365nm). Flow cytometry analysis of *E. coli* cells presenting ALFA-Pb without (-UV) and with (+UV) irradiation with light (λ=365nm) and incubation with sfGFP-ALFA-tag. Controls show uninduced cell population that have not expressed the ALFA-Pb (gray signal covered behind red signal).

MATERIALS AND METHODS

Recombinant gene expression and protein purification

Table S1 shows the list of nanobody and photobody proteins prepared in this study and their encoding expression plasmids. The amino acid sequences are listed below the Supporting Tables. *E. coli* BL21(DE3) Gold cells were transformed with the respective expression plasmid without amber stop codons for suppression in their encoding genes. For amber stop codon suppression *E. coli* K12(DE3) UT5600 cells[1b, 2] were used and were co-transformed with the respective expression plasmid and the pEVOL-ONBY plasmid. Cells were cultured at 37 °C in LB-medium (600 mL) with the corresponding antibiotic(s) (100 µg/mL ampicillin, 34 µg/mL chloramphenicol) until an OD600 of 0.7 – 0.9 was reached. The temperature was then shifted to 28 °C and protein expression was induced for 4 h by adding IPTG (0.4 mM final concentration). For suppression conditions, 0.5 mM o-(2-nitrobenzyl)-L-tyrosine (ONBY), presolved in 1 M NaOH, and 0.2% arabinose were added, and cells were incubated for 4 h at 37 °C. Cells were pelleted by centrifugation and resuspended in Ni-NTA buffer (50 mM Tris/HCl, 300 mM NaCl, pH 8.0). Resuspended cells were ruptured using sonification. Insoluble material was removed by centrifugation and the supernatant fractions were used to purify the proteins. Purification of His-tagged proteins *via* Ni-NTA affinity chromatography was performed at 4 °C using gravity flow columns with a bed volume of 1.0 mL of Ni-NTA resin (Cube Biotech) pre-equilibrated with Ni-NTA buffer with 20 mM imidazole. Following loading of the supernatant fractions (with 20 mM imidazole), three steps of washing with five to ten column volumes (cv) of Ni-NTA buffer (with 20 mM imidazole) were performed. Proteins were eluted with 4 mL Ni-NTA buffer (with 250 mM imidazole) fractions containing the desired protein were pooled and concentrated in Vivaspin® Turbo 4 concentrator spin columns (Sartorius, Göttingen, Germany) to ~¼ of the volume.

As a second purification step a size exclusion chromatography was performed on an ÄKTA Purifier System (GE Healthcare) with a Superdex200 column and a flow rate of 1 mL/min in PBS buffer (140 mM NaCl, 2.7 mM KCl, 1.5 mM KH_2PO_4, 8.1 mM Na_2HPO_4, pH 7.4). Protein elution was monitored by absorption at 280 nm and collected fractions were analyzed by SDS-PAGE. Purified fractions were combined, 10% glycerol (v/v) was added, concentrated in Vivaspin® Turbo 4 concentrator spin columns (Sartorius, Göttingen, Germany), flash frozen in liquid nitrogen and stored at -80 °C. Protein concentrations were determined using the calculated extinction coefficient at 280 nm.[1b]

We achieved a yield of 0.58 mg purified protein per liter of *E. coli* cell culture medium for the ALFA-Nb expressed in the periplasm. For suppression of ALFA-Pb we achieved 0.32 mg/L (55 % efficiency).

Protein bioconjugation with AlexaFluor647

Nanobody and photobody proteins were chemically modified using AlexaFluor647 maleimide (Invitrogen, Carlsbad, USA). A final concentration of 20 µM protein in PBS buffer was incubated with 50 µM TCEP for 15 min at RT before labeling to reduce potentially oxidized cysteines. AlexaFluor647 maleimide was then added in 5x excess and incubated for 30 min in the dark at RT. This step was repeated once. To quench the reaction 2 mM DTT was added and incubated for 10 min in the dark at RT. The labeled protein was purified by Ni-NTA affinity chromatography and concentrated using Vivaspin® Turbo 4 concentrator spin columns to respective concentrations.

Fluorescence and Coomassie-stained SDS-PAGE gel images

Fluorescence SDS-PAGE gel images were captured on a Typhoon FLA 9500 laser scanner (GE Healthcare) before Coomassie-staining. Fluorescence stage was used with a 635 nm laser and "Alexa Fluor 647"-method for detection. Coomassie-stained SDS-PAGE gel images were captured on a CanonScan 9000F Mark II system.

UV irradiation of cells using an LED lamp

For UV irradiation of cells before fixing with PFA an LED lamp was used (M365LP1-365 nm; Thorlabs Inc, Newton, USA). An LED power of 530 mW was determined for the instrument using a thermophile sensor (A3, P/N 7Z02621, Aperture: Ø 9.5 mm) (Ophir Optronics Solutions Ltd., Jerusalem, Israel). Samples were irradiated for the indicated time periods with an irradiation distance of 5 cm.

Nanobody display on cell surface

Nanobody encoding genes were cloned into a pBAD-vector for the AIDA autodisplay system[1, 3] using *E. coli* DH5α cells as cloning host. *E. coli* BL21 Gold (DE3) cells were co-transformed with the respective AIDA plasmid (Table S2) and, if applicable, with the pEVOL-ONBY plasmid for incorporation of o-(2-nitrobenzyl)-L-tyrosine (ONBY). Presenting *E. coli* cells were prepared by inoculating LB medium (20 mL) with respective antibiotics with an overnight culture in the ratio 1:20. Bacterial cell cultures were then grown at 37 °C with shaking (180 rpm) until an OD600 of 0.5 was reached. In case of suppression conditions, ONBY (3 mM final concentration) was added to the medium and gene expression was induced with 0.4% arabinose. Induced cells were cultured for 3 h at 30 °C in case of regular expression without amber stop codon suppression or for 2 h at 37 °C in case of suppression with the unnatural amino acid. The cells were then washed three times with ice cold PBS buffer (140 mM NaCl, 2.7 mM KCl, 1.5 mM KH_2PO_4, 8.1 mM Na_2HPO_4, pH 7.4; sterile-filtered) using a centrifugation step (5000 *g*) at 4 °C for 2 min to pellet the cells, and then stored after resuspension in 1 mL PBS buffer at 4 °C overnight.[1b]

Determination of binding affinity of nanobodies presented on cell surface by flow cytometry

Nanobody presenting *E. coli* cells were washed with ice cold PBS buffer (140 mM NaCl, 2.7 mM KCl, 1.5 mM KH_2PO_4, 8.1 mM Na_2HPO_4, pH 7.4; sterile-filtered), pelleted by centrifugation (5000 *g* for 2 min at 4 °C) and were then resuspended in PBS buffer and diluted to OD600 = 1.0. For photo-deprotection, 100 µL cells (OD600 = 1) were transferred into a thin-walled PCR tube (Greiner Bio-One, Kremsmuenster, Austria) and irradiated for 45 sec. For binding of sfGFP-ALFA-tag (**3**), a total volume of 100 µL cell suspension (OD600 = 1) were pelleted, resuspended in sfGFP-ALFA-tag solutions of different concentrations (each 40 µL) and then incubated for 20 min at 25 °C. For detection of the myc-tag, cells were resuspended in 50 µL PBS, 0.5 µL primary monochlonal anti-myc antibody (MA1-21316, Thermo Fisher) was added and incubated for 30 min at 25 °C. After washing two times with 200 µL PBS, cells were resuspended in 50 µL PBS and 0.5 µL secondary antibody anti-mouse-DyLight633 antibody (10006103; Thermo Fisher) was added and incubated for 20 min at 25 °C. Subsequently, 200 µL PBS was added and the cells were washed three times with 200 µL PBS each. Cells were stored in the dark on ice until flow cytometry analysis was carried out.

Prior to analysis by flow cytometry, the samples were diluted ten-fold with PBS buffer. Flow cytometry measurements were performed with a FACS Aria III (BD, Heidelberg, Germany) using a 488 nm laser for excitation and 530/30 BP- and 502 LP-filter for detection of GFP (698 V). For DyLight633 detection, a 633 nm laser was used for excitation in addition to a 660/20 BP filter detection (852 V). For each sample, a total of 50,000 cells were recorded at a rate of 2,000 cells per second and analyzed by FACS DIVA 8.0 software (BD, Heidelberg, Germany). The forward and sideward scattering was detected with 300 V. Measured mean fluorescence of samples were plotted against the log (concentration) of sfGFP-ALFA-tag and K_D determined by nonlinear fitting.[1b]

Microscale thermophoresis (MST)

A dilution series with 16 aliquots of the ALFA-photobody (**1**) was prepared in PBS buffer before measurement. 10 µL of fluorescent sfGFP-ALFA-tag protein (10 nM final concentration) was mixed with 10 µL of the 16 photobody aliquots and incubated for 15 min at 25 °C. MST measurements were performed on a Nanotemper Monolith NT.115 with standard coated capillaries (NanoTemper Technologies, Munich, Germany). The blue LED power was at 100% and the MST power was at 80%. Measured data points were exported and plotted against the log (concentration) of nanobody to determine K_D by nonlinear fitting.

Cell culture techniques and cell surface labeling

HeLa cells were cultured in EMEM (supplemented with 10% fetal calf serum, 1% non-essential amino acids and 1% L-glutamine) at 37 °C and 5% CO_2.

50% confluent cells were used for transient transfection with the plasmid encoding HA-ALFA-tag-Trx-TMD-mCherry using calcium phosphate precipitation technique on a 24 mm coverslip in a 35 mm cell

culture dish, followed by 16 to 20 h of incubation. Table S3 lists the construct used for transient transfections.
For labeling of the cell surface, cells were washed two times with PBS and 1 mL of a 10 nM solution of the AlexaFluor647 modified nanobody or photobody in EMEM was added. Incubation was performed at room temperature for 15 minutes.

Confocal laser scanning microscopy

Cells were fixed with 4% paraformaldehyde in PBS for 20 min at 25 °C, washed three times with PBS, stained with DAPI and mounted on coverslips using Aqua/Poly-Mount mounting solution (Polysciences). Confocal microscopy was carried out using a 63X water-immersion objective lens on a Leica DMi8 system. DAPI laser (405 nm), mCherry laser (552 nm), Alexa647 laser (638 nm).

Mass spectrometry

Mass analyses of intact proteins were performed using an UltiMate™ 3000 RS system (Thermo Fisher Scientific GmbH, Dreieich, Germany) connected to a maXis II UHR-qTOF mass spectrometer (Bruker Daltonik GmbH, Bremen, Germany) with a standard ESI source (Apollo, Bruker Daltonik GmbH, Bremen, Germany). Proteins were reduced with 2 mM TCEP at room temperature for 10 minutes to avoid inhomogeneity issues. Then, samples were acidified using a 5% formic acid solution to reach a pH 2-3 and centrifuged (14000 rpm, 3 min). According to the protein concentration, an appropriate volume of the supernatant was loaded on a C4 column (Advance Bio RP-mAb C4, 2.1 mm x 50 mm, 3.5 µm, Agilent Technologies, Waldbronn, Germany) at a flow rate of 0.6 mL/min in 5% eluent B (eluent A: 0.1% formic acid in water; eluent B: 0.1% formic acid in acetonitrile). After a desalting period of 7 minutes at 5% B, a steep gradient was applied (5-60% B in 2 min). MS settings: capillary voltage 4500 V, end-plate offset 500 V, nebulizer 5.0 bar, dry gas 9.0 L/min, dry T=200°C, mass range m/z 300-3000. Data were analyzed with DataAnalysis 4.4 (Bruker Daltonik GmbH, Bremen, Germany) and deconvolution was performed using the MaxEnt algorithm implemented in the software. Unless otherwise mentioned, the averagine-based SNAP algorithm was employed to identify peaks and to calculate the monoisotopic masses.

SUPPORTING TABLES

Table S1. List of purified recombinant nanobody constructs and their expression plasmids

Protein number	Name of construct	Encoding plasmid	Vector backbone
**1**	ALFA-Nb(Y42ONBY)-H_6	pBJ299 + pEVOL(ONBY)	pET22b(+)
**2**	ALFA-Nb-H_6	pBJ298	pET22b(+)
**3**	sfGFP-ALFA-tag-H_6	pBJ278	pET22a

Table S2. List of plasmids used for nanobody presentation in the AIDA autodisplay system

Protein number (when mentioned in text and figures)	Name of construct	Encoding plasmid	Vector backbone
**-**	ALFA-Nb-myc-AIDA	pBJ292	pBAD[3b]
**-**	ALFA-Nb(Y42ONBY)-myc-AIDA	pBJ296 + pEVOL(ONBY)	pBAD[3b]

Table S3. List of plasmids used for cell culture experiments

Protein number	Name of construct	Encoding plasmid	Vector backbone
**4**	HA-ALFA-tag-Trx-TMD-mCherry	pKSH18	pDisplay

Amino acid sequences of purified recombinant nanobody constructs

(1) ALFA-Nb(Y42ONBY)-H_6

MGCGGSGEVQLQESGGGLVQPGGSLRLSCTASGVTISALNAMAMGW**X**RQAPGERRVMVAA
VSERGNAMYRESVQGRFTVTRDFTNKMVSLQMDNLKPEDTAVYYCHVLEDRVDSFHDYWG
QGTQVTVSSLEHHHHHH

(2) ALFA-Nb-H_6

MGCGGSGEVQLQESGGGLVQPGGSLRLSCTASGVTISALNAMAMGWYRQAPGERRVMVAA
VSERGNAMYRESVQGRFTVTRDFTNKMVSLQMDNLKPEDTAVYYCHVLEDRVDSFHDYWG
QGTQVTVSSLEHHHHHH

SUPPORTING REFERENCES

[1] a) J. Jose, R. M. Maas, M. G. Teese, *J Biotechnol* **2012**, *161*, 92-103; b) B. Jedlitzke, Z. Yilmaz, W. Dorner, H. D. Mootz, *Angew Chem Int Ed Engl* **2020**, *59*, 1506-1510.

[2] a) J. Jose, S. Handel, *Chembiochem* **2003**, *4*, 396-405; b) J. K. Böcker, W. Dorner, H. D. Mootz, *Chem Commun (Camb)* **2019**, *55*, 1287-1290.

[3] a) S. Oloketuyi, C. Dilkaute, E. Mazzega, J. Jose, A. de Marco, *Appl Microbiol Biotechnol* **2019**, *103*, 4443-4453; b) S. Palei, K. S. Becher, C. Nienberg, J. Jose, H. D. Mootz, *Chembiochem* **2019**, *20*, 72-77.

APPENDIX

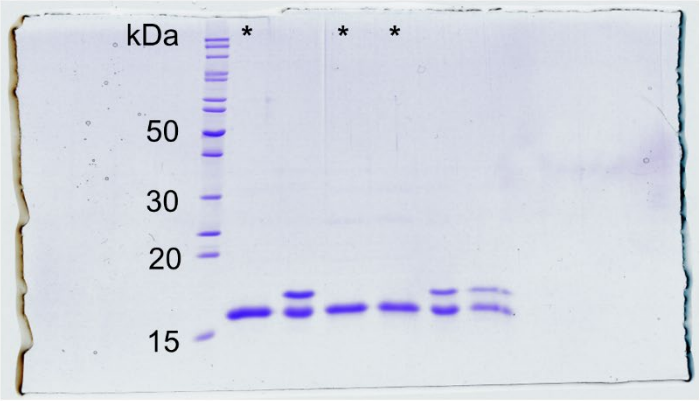

Figure A1. Uncropped SDS PAGE gel of image used in Figure 2A. The asterisk (*) labels the three used lanes in Figure 2A.
